# Tailoring of Optical Properties of Methacrylate Resins Enriched by HPHT Microdiamond Particles

**DOI:** 10.3390/nano12152604

**Published:** 2022-07-28

**Authors:** Ewelina Kowalewska, Mateusz Ficek, Krzysztof Formela, Artur Zieliński, Srinivasu Kunuku, Miroslaw Sawczak, Robert Bogdanowicz

**Affiliations:** 1 Faculty of Electronics, Telecommunications and Informatics, Gdańsk University of Technology, 11/12 G, Narutowicza St., 80-233 Gdansk, Poland; kowalews@agh.edu.pl (E.K.); matficek@pg.edu.pl (M.F.); srinivasu.kunuku@pg.edu.pl (S.K.); 2 Academic Centre for Materials and Nanotechnology, AGH University of Science and Technology, 30 A, Mickiewicza Ave., 30-059 Krakow, Poland; 3 Faculty of Chemistry, Gdańsk University of Technology, 11/12 G, Narutowicza St., 80-233 Gdansk, Poland; krzysztof.formela@pg.edu.pl (K.F.); artzieli@pg.edu.pl (A.Z.); 4 Centre for Plasma and Laser Engineering, The Szewalski Institute of Fluid Flow Machinery, Polish Academy of Science, 14 Fiszera St., 80-231 Gdansk, Poland; miroslaw.sawczak@imp.gda.pl

**Keywords:** methacrylate composites, microdiamond particles, polymer resin, optical properties, fluorescence

## Abstract

Diamond particles have great potential to enhance the mechanical, optical, and thermal properties of diamond–polymer composites. However, the improved properties of diamond–polymer composites depend on the size, dispersibility, and concentration of diamond particles. In the present study, diamond–polymer composites were prepared by adding the microdiamond particles (MDPs) with different concentrations (0.2–1 wt.%) into polymers (acrylate resins) and then subjected to a photocuring process. The surface morphology and topography of the MDPs–polymer composites demonstrated a uniform high-density distribution of MDPs for one wt.% MPDs. Thermogravimetric analysis was employed to investigate the thermal stability of the MDPs–polymer composites. The addition of MDPs has significantly influenced the polymers’ thermal degradation. Absorption and emission spectra of thin layers were recorded through UV/Vis spectrophotometry and spectrofluorimetry. The obtained results revealed a significant increase in the fluorescence intensity of MDPs–polymer composites (at 1 wt.% of MDPs, a 1.5×, 2×, and 5× increase in fluorescence was observed for MDPs–green, MDPs–amber daylight, and MDPs–red resin, respectively) compared with the reference polymer resins. The obtained results of this work show the new pathways in producing effective and active 3D-printed optical elements.

## 1. Introduction

Nanodiamonds (NDs) are allotropes of nanocarbons that are promising for numerous applications. NDs are widely used in electronics, energy storage, and coating manufacturing due to their unique physicochemical properties, such as high chemical stability, superior hardness, and thermal conductivity [1,2]. In addition, NDs’ chemical stability and biocompatibility properties are highly desirable in medical fields, including imaging, diagnosis, and targeted drug delivery [3,4,5,6,7]. ND powders can be obtained by various methods, including dynamic synthesis; high-pressure, high-temperature synthesis (HPHT); and chemical vapor deposition (CVD). NDs synthesized via the detonation method are frequently mentioned as helpful in preparing ND composites [8,9] by improving their mechanical properties [8,9]. Monocrystalline diamond powders produced by the HPHT method show high optical transparency for potential optoelectronic applications [10,11]. Moreover, structural defects and impurities responsible for the fluorescence of diamonds can be introduced deliberately or unintentionally during the material synthesis. Without expensive high-energy particle irradiation, HPHT diamonds exhibit synthesis-induced defects, such as nitrogen atoms, leading to form color centers for fluorescent emission [12,13,14].

Composites based on ND-incorporated polymer matrices have attracted significant attention due to their improved toughness, strength, transparency, and heat-resistance [15,16,17]. The large active surface area of small carbon nanostructures creates large interfacial areas in composites, and such regions exhibit better physical properties than pure polymers because of the increased interaction with nanoparticles [18]. Recent developments have been focused on the homogeneous distribution of NDs in the polymer matrices by surface functionalization of NDs, resulting in enhanced mechanical properties demonstrated for ND–polymer composites [5]. The most prominent polymers used in the synthesis of ND–polymer composites are poly(methyl methacrylate) (PMMA) [19,20], polyurethane [21], polypropylene [22], and epoxy [23,24]. An increased Young’s modulus and higher glass transition temperature have been observed in composites containing only 0.25 wt.% NDs in a polyurethane-based matrix [17]. ND–polymer composites have also exhibited potential in transparent UV filter coating applications [18,25]. Methacrylate oligomer composites were synthesized by 3D printing [26,27,28]. The obtained ND–polymer resins showed that the addition of NDs significantly reduced the contact angle of the resins, reduced sorption, and improved the mechanical properties. 

Zhang et al. reported an enhanced Young’s modulus and yielding stress of 85% and 15%, respectively, for a diamond nanothread (DNT)-incorporated PMMA matrix [29]. The DNT reinforcement mechanism is mainly based on mechanical interlocking and interfacial interactions, which are affected by the DNT’s morphology. Takada et al. reported on acrylic denture base resins modified with NDs (0.014–1 wt.%) [30], and the results showed that a small amount of NDs could impart desirable thermal and esthetic properties to denture base resins. In addition, the ND denture base composites demonstrated antibacterial properties [19,28]. A recent review by Kausar described many approaches to synthesizing ND–polymer composites and the impact of the NDs on the morphological, mechanical, or thermal properties [31]. Octadecylamine-functionalized ND–poly(l-lactic acid) composites have exhibited great potential for bone tissue engineering and regenerative medicine due to their excellent mechanical properties, intrinsic fluorescence, and biodegradability [32]. However, according to the best of our knowledge, less attention has been paid to improving the optical properties of polymers by incorporating diamond particles into the polymer matrix. Therefore, the present study intended to synthesize microdiamond particles (MDPs)–polymer composites with acrylate-based resins and investigate their thermal stability and optical properties. Furthermore, the effect of MDPs was evaluated in terms of the morphology and optical properties.

## 2. Materials and Methods

### 2.1. Materials

Monocrystalline diamond powder MSY 0–0.25 with a median grain diameter of 125 nm was purchased from Pureon AG (Lengwil, Switzerland). Diamond particles were produced by milling the monocrystalline diamond films synthesized by the HPHT process. Acetone was obtained from Sigma-Aldrich, Poznań, Poland. The acrylate-based resins (green daylight resin 3D firm, red daylight resin 3D firm, and amber daylight resin 3D hard) were purchased from Photocentric Ltd. (Peterborough, UK), and the properties of the resins are listed in Table 1. The resins are chemically composed of acrylate oligomers, acrylate monomers, methacrylate monomers, methacrylate oligomers, and photoinitiators. 

### 2.2. Fabrication of Composites

**Figure 1:**
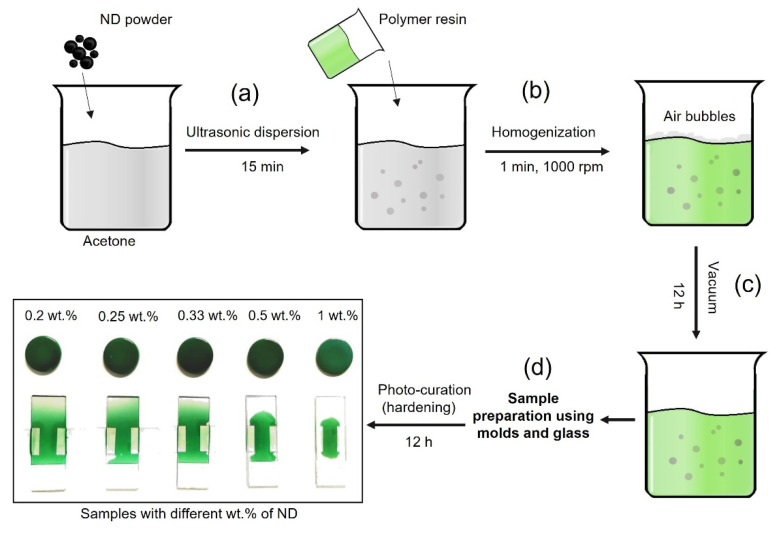
Schematic of the preparation of MDPs–polymer composites: dispersion of acetone and MDPs solution (**a**), homogenization of the obtained solution with polymer resin (**b**), air-bubble removal with vacuum (**c**), and photocuring of prepared samples (**d**).

MDPs–acrylate composites were prepared by adding 1% of MDPs added to the acetone and then subjected to ultrasonication for 15 min to obtain the proper dispersion of the MDPs throughout the acetone. The MDPs solution was then added to polymer resin in weight proportions of 1:1, 1:2, 1:3, 1:4, and 1:5, and these MDPs–acrylate composites were subjected to homogenization for one minute, at 1000 rpm, with a homogenizer (CAT × 120). This process led to obtaining MDPs–acrylate with different MDP concentrations: 0.2, 0.25, 0.33, 0.5, and 1 wt.%. The samples were placed in a vacuum oven for 12 h, at room temperature, to eliminate air bubbles (Figure 1). Thin layers of MDPs–acrylate composites (up to 245 µm) were prepared by pouring the liquid samples between two sodium glass slides and two horizontally located coverslips. Due to the presence of the photoinitiators, the abovementioned processing steps were carried out with minimal exposure to visible light. Subsequently, the samples underwent photocuring for 12 h. 

Shenderova et al. described that it was difficult to attain the required dispersion of NDs by direct mechanical mixing of the NDs into the PDMS matrix, which results in the formation of large (fractions of a millimeter) agglomerates of NDs in the ND–PDMS composite. An intermediate solvent was employed to resolve this issue; the solvent served as a dispersion medium for the nanoparticles before mixing with the polymer matrix [33]. Therefore, in this present study, acetone was used as an intermediate solvent to reduce the agglomeration of the MDPs. The entire process was repeated for each kind of acrylate resin. To evaluate the effect of the added acetone, one set of samples was prepared by adding the MDPs directly to the polymer resin, and pure resins were utilized as reference material.

### 2.3. Measurements

The morphology and topography of the MDPs–polymer composites were observed by employing a Scanning Electron Microscope (SEM) (Phenom XL, Waltham, MA, USA) with a backscattered electron detector, operating with the accelerating voltage of 10 kV. AFM topography images were obtained by using a NTEGRA (NT-MD, Moscow, Russia) system with NSG30 probes (TipsNano, Tallinn, Estonia), having geometric parameters of 125 × 48 × 4 μm with a tip curvature radius equal to 10 nm. The measurements were carried out in atmospheric conditions, with a moving sample, in semi-contact mode, and the set point was set to half the value of the free oscillation amplitude. Thermogravimetric analysis (TGA) of the MDPs–polymer composites was carried out with a TG 209 F3 apparatus from the Netzsch Group (Selb, Germany). The TGA process was performed for six samples at one time (approx. 10 mg weight), with the samples placed on a ceramic dish. The measurements were performed under a nitrogen atmosphere over a temperature range from 30 to 800 °C, with a heating rate of 10 °C/min.

Absorption spectra were obtained by using a double-beam UV-9000 Metash Spectrophotometer (Shanghai, China). The signal was registered over the 300–1000 nm range, with a 5 nm step. Two sodium glasses with the same thickness as the investigated samples were used as a reference beam. The transmittance of the samples was calculated through Beer’s law. The fluorescence spectra were recorded by utilizing a custom-built setup, using a 532 nm CW Nd:YAG SHG laser (Millenia, Spectra Physics, Milpitas, CA, USA) as an excitation source. The samples were excited by laser from the front surface, at 45 degrees. The fluorescence signal was collected by using a quartz lens and focused on the entrance of an optical fiber. A bandpass filter (OG570, Schott, NY, USA) was used in the detection path to block the laser radiation. The fluorescence signal was analyzed by using a 0.3 m monochromator (SR303i, Andor, Belfast, Great Britain) equipped with 600 groove/mm grating and recorded with an iCCD detector (DH740, Andor, Belfast, UK). 

## 3. Results and Discussion

### 3.1. Topology and Thermal Behavior

**Figure 2:**
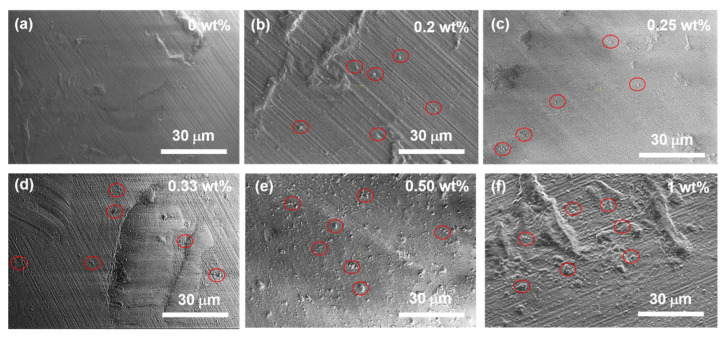
SEM images of the MDP composites based on green daylight resin with (**a**) 0 wt.% (green polymer), (**b**) 0.2 wt.%, (**c**) 0.25 wt.%, (**d**) 0.33 wt.%, (**e**) 0.5 wt.%, and (**f**) 1 wt.% of MDPs content (0 wt.% belongs to the pure polymer without MDPs). MDPs and MDP aggregates are marked with red circles.

The surface morphology of the MDPs–polymer composites was observed by using the SEM, and the obtained SEM images are shown in Figure 2. Figure 2a shows the surface morphology of the reference sample (MDPs 0 wt.%) derived from the polymer that depicts the river lines kind of surface feature with a smooth surface [34]. Initially, 0.2 wt.% of MDPs was added to the polymer, and the resulting surface morphology revealed an unevenly dispersed micron size MDPs agglomerates on the polymer’s lines kind of surface (Figure 2b). Next, the MDPs’ filler concentration was increased to 0.25% (Figure 2c), 0.33% (Figure 2d), and 0.50% (Figure 2e), and the obtained surface morphology images demonstrate that the uniform distribution of MDPs in the polymer matrix has been increased with the concentration of MDPs. The increase in MDPs’ concentration from 0.2 to 0.25 wt.% has not shown a significant rise in MDPs’ distribution in the polymer matrix (Figure 2c). However, the river-lines pattern of the polymer surface has been partially covered with MDPs for the MDP-filler concentration of 0.33 wt.% (Figure 2d). The distribution of MDPs has been increased and clearly shows the river-lines pattern of the polymer surface MDP agglomerates for the MDP-filler concentration of 0.50% (Figure 2e). Figure 2f shows the SEM morphology of 1% of MDPs added to the polymer composite, revealing a high density of MDP agglomerates with sizes up to 2 µm. Therefore, an MDP concentration up to 0.50% added to the polymer significantly improved a uniform distribution of MDPs throughout the polymer. A further increase in the concentration of MDPs added to polymer led to the formation of high-density MDP aggregates on the MDPs–polymer surface. The MPD aggregates that appeared on the polymer composite’s surface were formed during the hardening process. MDPs and MDP aggregates are marked in red circles in the SEM images of MDPs–polymer composites (Figure 2a–f). The distribution of MDPs in the polymer matrix is consistent with other publications [35].

MDPs–polymer composites were prepared with and without acetone for the MDP filler concentration of 1% to investigate the role of acetone in mixing or distributing the MDPs in the polymer. The surface morphology of the MDPs–polymer composite with the MDPs concentration of 1% in acetone depicts a uniform distribution of MDPs with sizes ~1–3 µm (Figure 2f). In contrast, the MDPs–polymer composite containing the MDP concentration of 1% and without the addition of acetone leads to the formation of large MDP agglomerates with sizes of 10 µm (Figure not shown). It has been reported that direct mixing of MDPs into the polymer resulted in the formation of large aggregates due to a lack of dispersion, and an intermediate medium is required to achieve appropriate dispersion of MDPs throughout the polymer matrix [33]. Therefore, in the present study, acetone was used as an intermediate medium to disperse the MDPs and to form a homogeneous arrangement of MDPs in the resin matrix. In addition, the surface morphology of the MDPs–polymer composite changed with the concentration of MDPs [36,37]. Therefore, the solvent was determined to be necessary to obtain homogeneous samples, and further measurements were conducted only on the acetone-based materials.

**Figure 3:**
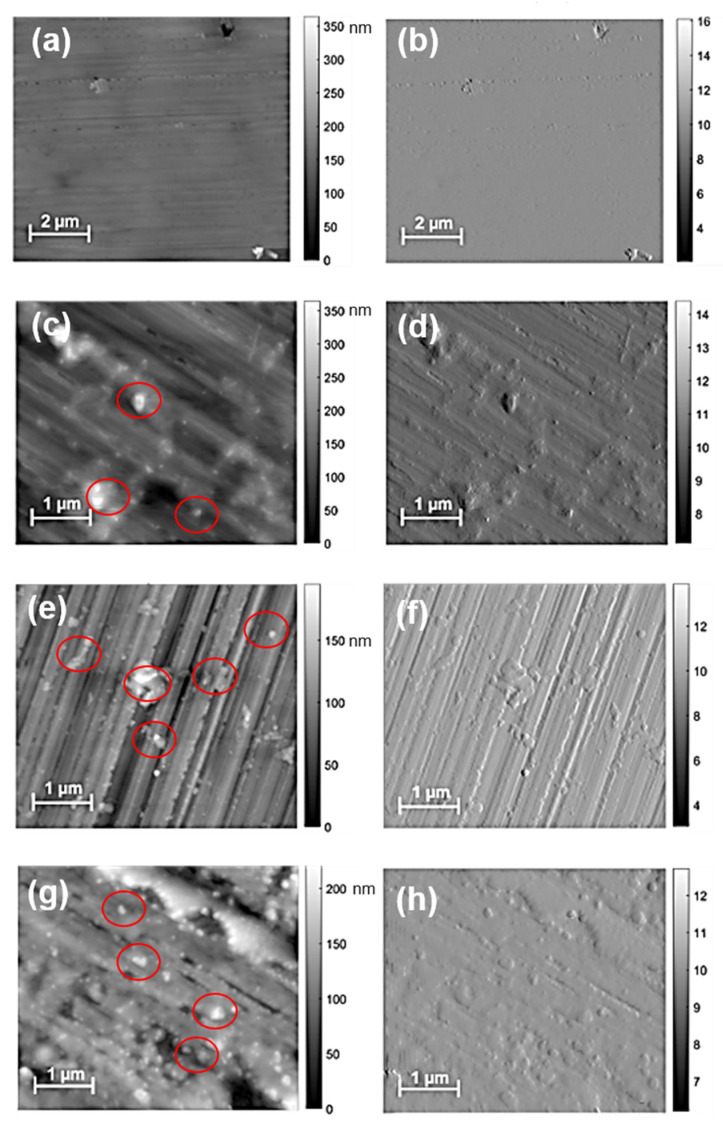
AFM topography and amplitude images of the pure polymer sample (**a**,**b**), MDPs–red resin composite with 1 wt% MDPs (**c**,**d**), MDPs–green resin composite with 1 wt% MDPs (**e**,**f**), and MDPs–amber resin with 0.5 wt% MDPs (**g**,**h**).

The topography and amplitude of different MDPs–polymer composites are shown in Figure 3. Figure 3a,b shows the reference (without MDPs) resin sample’s AFM topography and amplitude image. The topography image reveals the polymer surface without any MDPs particles and river-lines kind of surface feature, which is similar than the SEM morphology of the polymer surface (Figure 2a). The topography images of MDPs–red resin (Figure 3c), MDPs–green resin (Figure 3e), and MDPs–amber resin (Figure 3g) composites revealed the presence of MDP agglomerates of 1 µm in size, irrespective of the concentration of MDPs. In addition, the MDPs are densely covered on lines of polymer surface for the filler concentration of 0.5 and 1 wt.%. MDPs and MDP aggregates were marked in red circles in the AFM topography images of MDPs–polymer composites (Figure 3). 

Moreover, MDPs clusters were found in a cross-section (Figure not shown) and the surface of the MDPs–polymer composites at a height of 200–350 nm. Additional amplitude imaging with the use of AFM enabled the imaging of nanostructures and their locations concerning the roughness of the surface of the base material, which is visible at smaller scales. The corresponding AFM amplitude images are labeled as Figure 3b for the reference sample, Figure 3d for MDPs–red resin, Figure 3f for MDPs–green resin, and Figure 3h for MDPs–amber resin composites. These images illustrate the momentary changes in the probe oscillations, allowing for more precise assessment of the locations of topography changes without the possibility of directly estimating the height. The tendency to agglomerate in the areas of primary surface unevenness is visible, especially in the case of higher MDP concentrations. 

**Figure 4:**
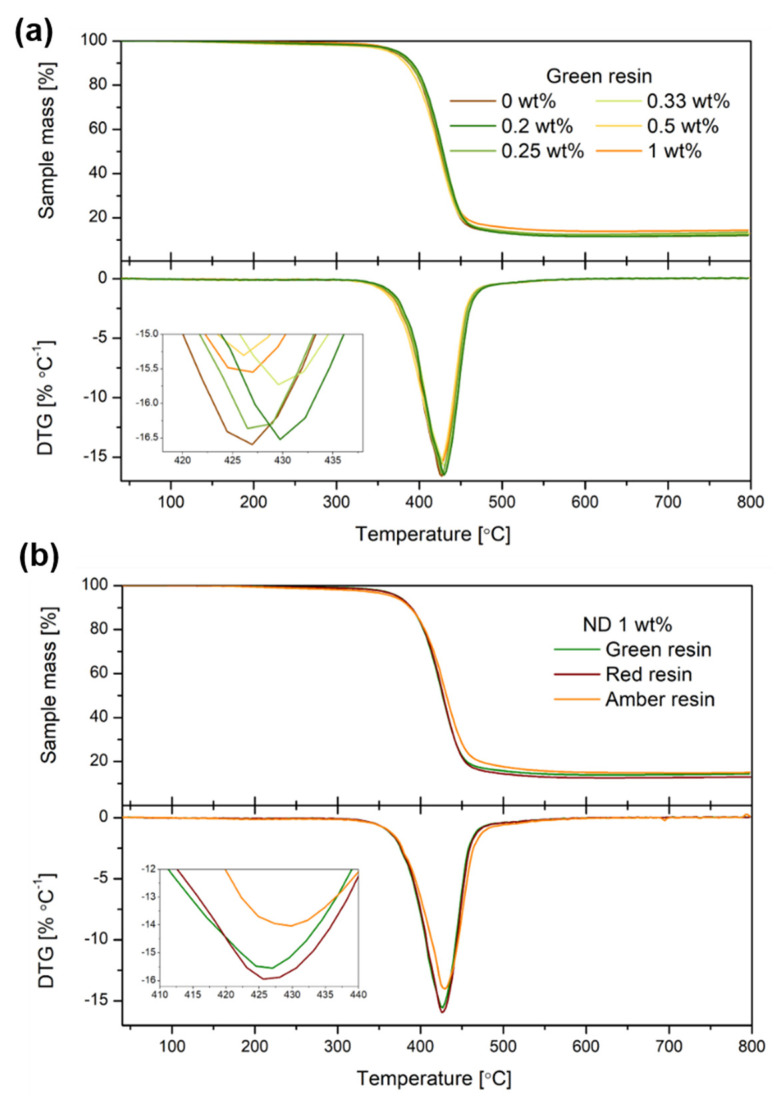
TG and DTG curves of MDPs–polymer composites with (**a**) green resin and (**b**) all samples with 1 wt.% (0 wt.% in Figure 4a belongs to the pure polymer without MDPs).

The upper part of Figure 4a shows the TGA curves of the MDPs–green resin samples, while their derivatives (DTG curves) are illustrated in the lower half. A significant loss in weight was observed for MDPs–green resin composites at temperatures between 350 and 550 °C, and it is attributed to the degradation of the polymer matrix. The degradation temperature (T_D_) ranged from 364.4 to 375.2 °C (Table 2). The lowest T_D_ value was obtained for the reference polymer sample, while MDPs samples have illustrated the T_D_ ~ 500 to 800 °C [see the supporting information Figure S1a]. A small increase in T_D_ was observed along with the increasing MDPs-filling concentration, which is related to the interaction of the MDPs with the polymer matrix and obvious higher thermal stability observed for the MDPs–polymer composite compared to the polymeric matrix. The difference between the T_D_ value of the reference sample and MDPs–green resin with 1 wt.% of ND was around 4 °C, which is similar to PMMA:MDPs composites (T_D_~6 °C) [34]. 

In addition, the degradation of the different MDPs–polymer composites (1 wt.% of MDPs), such as MDPs–green, MDPs–red, and MDPs–amber daylight resins, was studied. The obtained TGA and DTG curves of these samples are shown in Figure 4b. The upper part of Figure 4b shows the TGA curves of the MDPs–polymer resins, which depict the degradation of the polymer from 350 to 450 °C. The TD values of the MDPs–green and MDPs–red resins are 373.5 and 373.0 °C, respectively, while a lower TD value of 368 °C characterized the MDPs–amber daylight resin composite. According to the manufacturer’s specifications, amber resin is characterized by higher hardness and lower flexibility than the green and red polymers due to its slightly different chemical composition. Consequently, its interfacial interactions with the MDP filler lead to the maximum decomposition rate.

To better understand the thermal stability of the materials studied in the present study, the obtained results were compared with observations described by different research groups working in the field of polymer/diamond composites (Table 3). The comparative study in Table 3 shows no simple correlation between a higher concentration of MDP filler and improvement of the thermal stability of MDPs–polymer composites. It should be highlighted that many factors might affect the thermal stability of MDPs–polymer composites, such as the MDPs’ reinforcement filler content or characteristics (purity, surface area, particle size, etc.), polymer matrix composition (kind of polymer, its molecular weight or crosslink density), interfacial interactions in the matrix-filler system, and thermal stability measurements’ conditions. 

### 3.2. Optical Properties

**Figure 5:**
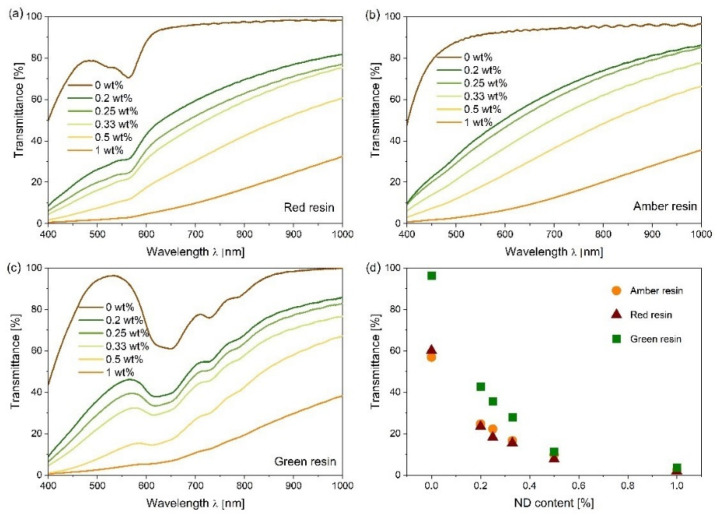
UV–Vis spectra of MDPs–polymer composites with amber resin (**a**), red resin (**b**), green resin (**c**), and comparison of the samples’ transmittance at 532 nm (**d**) (0 wt.% in above figures belongs to the pure polymer without MDPs).

The UV–Vis spectra of the MDPs–polymer composites are presented in Figure 5. The transmittance curves of different amounts of MDP filler added to MDPs–red resin (Figure 5a), MDPs–amber resin (Figure 5b), and MDPs–green resin (Figure 5c) composites demonstrate that the transmittance decreased with the increasing MDPs concentration. The influence of MDPs on the UV–Vis transmittance of the MDPs–polymer composites was confirmed [42,43,44]. The decrease in transmittance with the increase in the concentration of MDPs particles is caused by the different refractive indexes of the MDPs particles and the polymer matrix [45]. In addition, the scattering and reflection of incident light from the widely dispersed MDPs on the river-lines structure of the polymer surface (Figure 2 and Figure 3) are also factors for the decrease in transmittance for MDPs–polymer composites. However, in the case of amber daylight resin, the transmission value is relatively constant at 90% throughout the low-energy part of the spectrum (Figure 5d). As a result, the MDPs–polymer composites exhibited lower transmittance values at lower wavelengths.

Nevertheless, the transmittance increased with the wavelength. The observed high absorbance at lower wavelengths is mainly related to Rayleigh light scattering, which increases proportionally with the decrease in wavelength to 1/λ^4^. In addition, the MDPs–polymer composites exhibited strong absorption in the UV range (up to 380 nm) due to the presence of sodium glass. It was not possible to detect the signal in the higher-energy region. However, previous studies suggest the existence of the additional absorption band even below 300 nm [18]. In addition, the spectral characteristics of MDPs–polymer composites also depend on the size of the MDPs used in the preparation. Larionova et al. reported that the diameter of the diamond particles influences the color of the nanodiamond suspensions [46].

**Figure 6:**
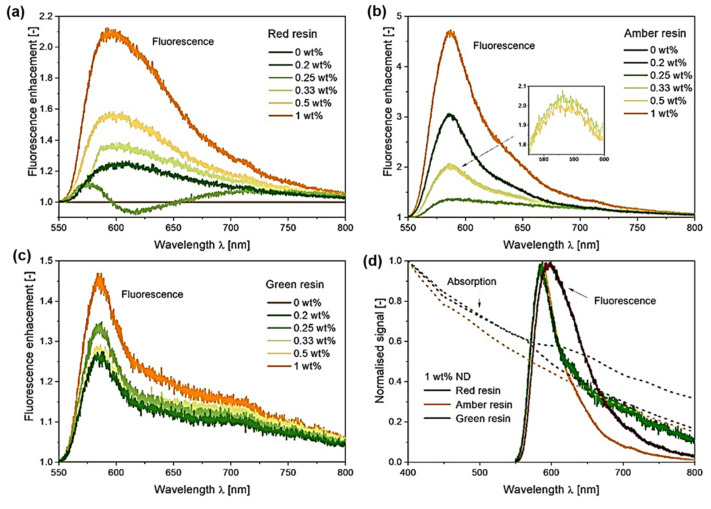
Fluorescence enhancement of MDPs–polymer composites with amber resin (**a**), red resin (**b**), and green resin (**c**). Graph (**d**) shows normalized fluorescence and absorption signal for composites with 1 wt.% ND (0 wt.% in above figures belongs to the pure polymer without MDPs).

The fluorescence spectra of the MDPs–polymer composites with different MDP concentrations are shown in Figure 6a for MDPs–red resin, Figure 6b for MDPs–amber resin, and Figure 6c for MDPs–green resin composites. All of the MDPs–polymer composites show a wide fluorescent band at around 588 nm and a small band at 700 nm, which is not observed in the case of the reference resin sample. The fluorescence spectra of the reference resin samples depict broad and relatively weak fluorescence, while the fluorescence signal increases significantly with the addition of MDPs to the polymers. The fluorescence of MDPs samples revealed the typical NV emission [see the supporting information Figure S1b], which is the origin for enhanced fluorescence from MDPs added polymers. It can be observed that the amber daylight resin demonstrated high emission intensity ranging from 560 to about 700 nm (Figure 6b). The MDPs–polymer composites showed an enhancement in fluorescence of the polymer at 588 nm. The fluorescence intensity increased monotonically with the MDPs concentration, and MDPs–polymer composites with the highest concentration of MDPs (1 wt.%) illustrated 4.5 times higher fluorescence intensity than the reference composite (Figure 6b). Therefore, the obtained results indicate that the fluorescence of MDPs arise from synthesis-induced defects such as NV centers. Moreover, the fluorescence of the MDPs is not quenched by the polymer and is responsible for a significant contribution to the emission signal of the MDPs–polymer composite. The addition of 1% of MDPs to the pure green resin resulted in a 1.5× increase in the intensity of the composite (Figure 6c). 

No significant fluorescence enhancement was observed for the MDPs–polymer composites with less than 1% MDPs. The MDPs–red composites demonstrated a slightly different emission spectrum than the other samples (Figure 6a,d). The main fluorescent band was shifted toward longer wavelengths and occurred around 600 nm, and for the addition of 1 wt.% MDPs, the fluorescence increased two times compared to the reference sample (Figure 6a,d). The differences in emission spectra shapes are related to the individual resin compositions, but the most interesting effect is the fluorescence enhancement observed for the MDPs–resin composites. Surface-enhanced fluorescence was initially observed for fluorophores under a strong electromagnetic field near metal nanoparticles. This effect is strongly observed in the resonance absorption of plasmonic metal nanoparticles. In this case, the fluorescence enhancement can be explained by the increased absorption and scattering cross-section of plasmonic nanoparticles and decreased fluorescence lifetime of the fluorophore that allows the excited state to return to the ground state at a higher frequency. Recent studies of core/shell-type nanoparticles show that the refractive index of the nanoparticle surrounding (core) has a critical role in fluorescence enhancement, and a significant fluorescence enhancement factor can be achieved by utilizing shells having a higher refractive index [47]. It can be assumed that, in the case of polymeric materials such as epoxy resin, local changes in the polymer structure caused by the presence of nanoparticles will also affect the intensity of fluorescence enhancement [48,49]. The effect of surface-enhanced fluorescence can be observed not only in the case of plasmonic metal nanoparticles but also in the case of non-metallic nanomaterials, such as ZnO nanoparticles and crosslinked polymer nanoparticles [50,51]. The results show that adding the most commonly used NV–NDs or HPHT–MDPs could be successfully applied in fluorescence studies and advanced optical elements. 

## 4. Conclusions

MDPs–polymer composites synthesized by the photocuring method were prepared by adding different concentrations (1%, 0.5%, 0.33%, 0.25%, and 0.2%) of MDPs to three kinds of acrylate resins. SEM morphological studies of the MDPs–resin composites confirmed the presence of MDPs in the polymer matrix, and the distribution density increased with the MDPs concentration. In addition, the AFM topography images of the MDPs–polymer composites are consistent with the SEM images. The density of the MDP agglomerates was also shown to increase with the increasing MDP filler concentration. Acetone was used as an intermediate solvent to prevent aggregation and to improve the dispersibility of the MDPs. TGA studies demonstrated that the stability of MDPs–polymer composites could be maintained up to around 360 °C, and it was found that low concentrations of MDPs have little influence on the degradation temperatures. Moreover, the fluorescence of the MDPs is not completely quenched by the polymer matrix. Importantly, no straightforward correlation was found between an increased concentration of MDP filler and the improved thermal stability of the prepared composites.

UV/Vis spectrophotometry made it possible to determine the ranges of wavelengths with reduced light transmission due to limited MDPs dispersion. Furthermore, intrinsic structural defects in the MDPs showed an influence on the MDPs–polymer composite’s absorption and emission spectra. The obtained results demonstrate the significant potential of the application of polymer–diamond composites in 3D-printed optical elements. An undoubted advantage of the materials described above is the uncomplicated synthesis process, which does not require expensive equipment. It was also proved that a significant increase in the fluorescence of the composites is achievable even at low concentrations of ND (<1%). Moreover, the HPHT–MDPs showed significant fluorescence from nitrogen-vacancy centers; this factor influences the emission properties of MDPs-based polymer composites. Subsequent work should focus on techniques to prevent the agglomeration of the nanodiamonds and improve their dispersion throughout the polymer matrix, which may affect higher optical transmittance of the composites. 

## Figures and Tables

**Table 1:** Mechanical properties of the resins according to the manufacturer.

Resin Type	Hardness	Tensile Strength	Elongation at Break	Viscosity (25 °C)	Volumetric Shrinkage
Green Daylight Resin 3D firm	65 Shore D	26 MPa	14.9%	560 cps	5.9%
Red Daylight Resin 3D firm	65 Shore D	26 MPa	14.9%	560 cps	5.9%
Amber Daylight Resin 3D hard	77 Shore D	42 MPa	8.7%	230 cps	6.6%

**Table 2:** TGA results of MDPs–polymer composites: TD—degradation temperature at which mass loss reaches 5%, T_max_—temperature corresponding to a maximum rate of decomposition, mr—weight percentage of the residue at 800 °C.

Resin Type	ND Content (wt.%)	T_D_ (°C)	T_max_ (°C)	m_r_ (%)
Green	0	369.4	426.4	12.1
Green	0.2	375.2	429.9	12.2
Green	0.25	371.7	427.5	13.4
Green	0.33	372.2	430.1	12.7
Green	0.5	364.4	426.3	13.0
Green	1	373.5	426.0	14.1
Red	1	373.0	426.3	13.0
Amber	1	368.0	430.0	15.0
Average calculated for all studied samples		370.9 ± 3.5	427.8 ± 1.9	13.2 ± 1.0
Average calculated for samples with 1 wt.% ND		371.5 ± 3.0	427.4 ± 2.2	14.0 ± 1.0

**Table 3:** Comparison of TGA for polymer/diamonds composites.

Sample Composition	TGA Conditions	T_D_ (°C)/MDPs Concentration	Observations	References
MDPs–acrylate composites MDPs content: 0.2–1.0 wt.%	30–800 °C (10 °C/min) N2	369.4 (0) 375.2 (0.2) 371.7 (0.25) 372.2 (0.33) 364.4 (0.5) 373.5 (1.0) 373.0 (1.0) 368.0 (1.0)	The overall trend is that a higher content of MDPs improved the thermal stability. However, in selected samples, deviations from this trend were observed. This may be related to chemical reactions between the MDPs and PMMA during synthesis that will affect the curing kinetics and consequently the performance properties of the studied materials.	This work
Poly (methyl methacrylate)/nanodiamond nanocomposites ND content: 0.1–1.0 wt.%	50–450 °C (10 °C/min) N2	287 (0) 288 (0.1) 289 (0.5) 293 (1.0)	The thermal stability increased proportionally to the ND content. A higher content of ND resulted in a higher T_-5%_.	[36]
Nanodiamond-attached exfoliated hexagonal boron nitride/epoxy nanocomposites Filler content: 10–50 phr	30–900 °C (10 °C/min) N2	PDT (polymer decomposition temperature) 378.3 (0) 389.2 (10) 401.8 (20) 398.2 (30) 392.3 (40) 391.2 (50)	For a sample with 10 phr of NDs, an increase of the decomposition temperature by 10.9 °C was observed. Moreover, as could be expected, the char residue for the sample with 10 phr of ND is much higher than it is for unadulterated epoxy resin. Similar trends were also observed in the other composites, which contained 20, 30, 40, and 50 phr of the EBN and NDEBN.	[38]
Epoxy/nanodiamond composites ND content: 0.1–1.0 wt.%	No information about temperature measurement range, (10 °C/min) Air and N2	Air N2 295 (0) 342 (0) 316 (0.1) 344 (0.1) 326 (0.5) 338 (0.5) 310 (1.0) 343 (1.0)	Interestingly, the sample with 0.5 wt.% of NDs decreased in thermal stability (in nitrogen) compared to the reference material. A similar tendency was observed in our present work.	[39]
Aminated nanodiamonds (A-NDs) as nanofillers in biological-grade acrylate-based 3D-printed materials ND content: 0.1 wt.%	100–800 °C (10 °C/min) N2	In the reference sample, 40% weight loss was recorded at 405 °C, while in the ND- and A-ND-incorporated nanocomposites, the same was observed at 420 and 426 °C, respectively.	The weight loss axis is presented only to 40%; therefore, it is difficult to comment on the presented results. The authors mentioned that the addition of ND increased the thermal stability of the polymer matrix. A very high level of residue for the reference sample indicated the presence of other fillers in the material.	[26]
ND-grafted poly(styrene) (ND-PS) and ND-grafted poly(methyl methacrylate) (ND-PMMA)	30–1000 °C (10 °C/min) N2	The authors presented only TGA curves. There is no additional information with a summary of thermal degradation parameters.	Chemical grafting of polymers to the surface of the NDs resulted in deterioration of thermal stability of the studied systems, which is related to the obvious lower thermal stability of polymers compared to the ND filler. TGA//DTG method is a useful tool for determining the grafting degree. For the studied systems, the grafting ratio of the PMMA was 10.56%, while for PS, it was 6.21%.	[40]
Various kinds of polymer chains (e.g., polystyrene, polymethyl methacrylate, and polyglycidyl methacrylate) were chemically grafted onto the deagglomerated nanodiamond by the wet-stirred-media-milling process	25–500 °C (10 °C/min) N2	The authors presented only TGA curves. There is no additional information with a summary of thermal degradation parameters.	Chemical grafting of polymers to the surface of the NDs resulted in deterioration of thermal stability of the studied systems, which is related to the obvious lower thermal stability of polymers compared to the ND filler. The grafting degrees for the studied systems were as follows: ~24% for PS, ~31% for PMMA, ~34% for PGMA.	[41]
PMMA + nanodiamonds ND content: 1–2 wt.%	30–1000 °C (20 °C/min) N2	The authors presented only TGA curves. There is no additional information with a summary of thermal degradation parameters.	The authors pointed out that a noticeable effect can be observed after high polymer weight loss. The results demonstrate that, even at relatively low dispersity and agglomeration of NDs in PMMA, the thermal degradation temperature can be increased.	[33]

## Data Availability

Data underlying the results presented in this paper are not publicly available at this time but may be obtained from the authors upon reasonable request.

## Supplementary Materials

The following supporting information can be downloaded at: https://www.mdpi.com/article/10.3390/nano12152604/s1, Figure S1: (a) TG curve of MDPs measured in air, which revealing the significant material loss between 500 °C–800 °C. (b) Photoluminescence spectrum of MDPs used as filler in the NDs-polymer matrix depicting the typical NV luminescence.
