# Supplementary material for: Tailoring of Optical Properties of Methacrylate Resins Enriched by HPHT Microdiamond Particles
Source: Nanomaterials (Basel). 2022 Jul 28;12(15):2604. doi: 10.3390/nano12152604 (PMC9370547; doi:10.3390/nano12152604)
Supplement: Supplementary file 1 [file nanomaterials-12-02604-s001.zip › nanomaterials-1775178-supplementary.pdf]

nanomaterials

# Supplementary Materials

# Tailoring of Optical Properties of Methacrylate Resins Enriched by HPHT Microdiamond Particles

**Ewelina Kowalewska [1,2], Mateusz Ficek [1], Krzysztof Formela [3], Artur Zieliński [3], Srinivasu Kunuku [1], Miroslaw Sawczak [4] and Robert Bogdanowicz [1,*]**

[1] Faculty of Electronics, Telecommunications and Informatics, Gdańsk University of Technology, 11/12 G, Narutowicza St., 80-233 Gdańsk, Poland; kowalews@agh.edu.pl (E.K.); matficek@pg.edu.pl (M.F.); srinivasu.kunuku@pg.edu.pl (S.K.)
[2] Academic Centre for Materials and Nanotechnology, AGH University of Science and Technology, 30 A, Mickiewicza Ave., 30-059 Kraków, Poland
[3] Faculty of Chemistry, Gdańsk University of Technology, 11/12 G, Narutowicza St., 80-233 Gdańsk, Poland; krzysztof.formela@pg.edu.pl (K.F.); artzieli@pg.edu.pl (A.Z.)
[4] Centre for Plasma and Laser Engineering, The Szewalski Institute of Fluid Flow Machinery, Polish Academy of Science, 14 Fiszera St., 80-231 Gdańsk, Poland; miroslaw.sawczak@imp.gda.pl
* Correspondence: rbogdan@eti.pg.gda.pl

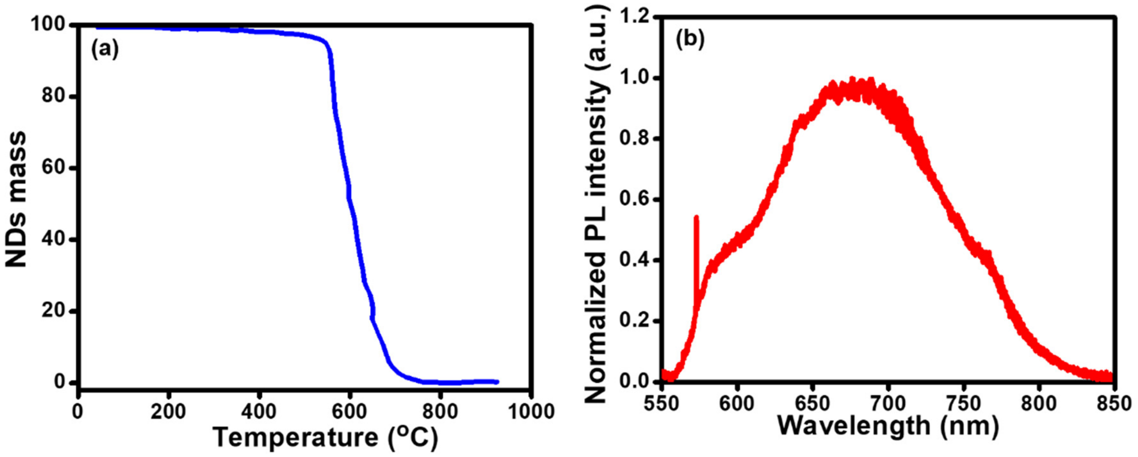


**Figure S1.** (**a**) TG curve of MDPs measured in air, which revealing the significant material loss between 500 °C – 800 °C. (**b**) Photoluminescence spectrum of MDPs used as filler in the NDs-polymer matrix depicting the typical NV luminescence. .
